# Training Set Construction for Genomic Prediction in Auto-Tetraploids: An Example in Potato

**DOI:** 10.3389/fpls.2021.771075

**Published:** 2021-11-24

**Authors:** Stefan Wilson, Marcos Malosetti, Chris Maliepaard, Han A. Mulder, Richard G. F. Visser, Fred van Eeuwijk

**Affiliations:** ^1^Biometris, Wageningen University & Research, Wageningen, Netherlands; ^2^Plant Breeding, Wageningen University & Research, Wageningen, Netherlands; ^3^Wageningen University & Research, Animal Breeding and Genomics, Wageningen, Netherlands

**Keywords:** training set construction, potato, sampling technique(s), genomic prediction (GP), auto-tetraploid

## Abstract

Training set construction is an important prerequisite to Genomic Prediction (GP), and while this has been studied in diploids, polyploids have not received the same attention. Polyploidy is a common feature in many crop plants, like for example banana and blueberry, but also potato which is the third most important crop in the world in terms of food consumption, after rice and wheat. The aim of this study was to investigate the impact of different training set construction methods using a publicly available diversity panel of tetraploid potatoes. Four methods of training set construction were compared: simple random sampling, stratified random sampling, genetic distance sampling and sampling based on the coefficient of determination (CDmean). For stratified random sampling, population structure analyses were carried out in order to define sub-populations, but since sub-populations accounted for only 16.6% of genetic variation, there were negligible differences between stratified and simple random sampling. For genetic distance sampling, four genetic distance measures were compared and though they performed similarly, Euclidean distance was the most consistent. In the majority of cases the CDmean method was the best sampling method, and compared to simple random sampling gave improvements of 4–14% in cross-validation scenarios, and 2–8% in scenarios with an independent test set, while genetic distance sampling gave improvements of 5.5–10.5% and 0.4–4.5%. No interaction was found between sampling method and the statistical model for the traits analyzed.

## Introduction

The utilization of DNA marker information for selection in breeding programs has increased over the last two decades and can be attributed to two factors: the decrease of genotyping costs, and the advances in quantitative genetics methodology. Genomic prediction (GP) is an example of one such methodological breakthrough that estimates breeding or genotypic values (depending on the application) by regressing known phenotypes against high density molecular markers (Meuwissen et al., 2001). GP allows the prediction of phenotypes from marker information which speeds up the breeding cycle, as the performance of new material can be assessed prior to phenotype expression (Heffner et al., 2010).

The potential genetic gains from GP hinge on its ability to predict phenotypes accurately. This prediction accuracy is dependent on various factors including but not restricted to: trait heritability (Heffner et al., 2009), statistical models (de los Campos et al., 2013), genetic architecture of traits (Daetwyler et al., 2013), population structure (Asoro et al., 2011; Guo et al., 2014) as well as the size and composition of the training/calibration set (Pszczola et al., 2012; Rincent et al., 2012; Bustos-Korts et al., 2016; Akdemir and Isidro-Sanchez, 2019). This study focuses on the composition of the training set; those individuals with both phenotype and genotype information, that are used to train the model and estimate the marker effects used to make future predictions. Having both input and target information, the training provides the necessary data so that statistical models can learn and estimate the relationship between explanatory variables and the target (James et al., 2013). The training set should be constructed in a way that it covers a space which closely resembles the space occupied by future test sets. This is important for GP because in more recent times, due to relatively cheap genotyping, molecular marker information (explanatory variables), can often be collected more efficiently than phenotype information (target). The question is, which individuals should be phenotyped and thus be used to calibrate the model and generate reliable predictions for individuals without phenotypic information?

Various sampling strategies are available for training set construction. Simple random sampling allows each individual in the population an equal probability of being in the training set and does not utilize any prior information regarding the material. If population structure exists and the material is separated into sub-populations, this information can be included in a sampling method known as stratified sampling. Stratified random sampling selects individuals based on their sub-population membership. Studies in diploids have shown that this method is superior to simple random sampling, although the improvement depends on the extent of the separation between sub-populations (Isidro et al., 2015). When there is little population structure, uniform coverage of the genetic space may be more suitable, and this is achieved with genetic distance sampling (Jansen and van Hintum, 2007). This methodology was first introduced to define core collections for germplasm banks, but the principle can be extended to construction of the training set, because similar to core collections, the objective is to obtain a subset of individuals that contain the genetic diversity present in a larger population. Rincent et al. (2012) proposed another method for sampling the training set that evaluates the quality of prediction for a set of genotypes. An algorithm was developed that chooses a training set that maximizes prediction accuracy, based on prediction error variance (PEV) and coefficient of determination (CD) measures (Rincent et al., 2012).

Numerous comparative studies have evaluated different methods of training set construction (Asoro et al., 2011; Isidro et al., 2015; Bustos-Korts et al., 2016; Akdemir and Isidro-Sanchez, 2019). These past studies have been conducted on diploids (2 copies of each chromosome) whereas in this study, the focus is on tetraploids (4 copies of each chromosome). Plants often exhibit polyploidy, as seen in potato (*Solanum tuberosum*), which is an auto-tetraploid and the subject of this article. There is potential for genetic gain in applying genomic prediction to potato (Slater et al., 2016), and this was put into practice in recent studies (Habyarimana et al., 2017; Sverrisdóttir et al., 2017; Endelman et al., 2018). The current study seeks to investigate the first step of GP not emphasized in the aforementioned papers, which is the impact of training set construction on GP accuracies in tetraploid potato. A secondary aspect of this study is the investigation of genetic distance measures, as these will be required to implement genetic distance sampling. Various measures of genetic distance exist, and the effect it has on selection accuracy has not yet been evaluated. There are some proposed measures that are allegedly more suitable for polyploids by accounting for allele dosage in polyploid heterozygotes, and by considering the presence of unknown alleles, where the absence of one allele does not necessarily imply presence of the other (Dufresne et al., 2014).

To ensure that the training set construction method would be robust for many GP models, three types of statistical models were assessed to generate prediction accuracies. They belong to three general categories of GP models: no marker selection, marker selection and models that capture non-additive effects. This was included in the study to investigate the presence/absence of a relationship between the sampling method for constructing the training set and the statistical model. The aim is to uncover the most suitable method for constructing the training set when GP for tetraploids is performed, and whether suitable methods exhibit codependencies with other influences including statistical model, sample size and trait architecture.

## Materials and Methods

### Plant Materials

Phenotypic and genotypic data were collected and made publicly available by The Solanaceae Coordinated Agricultural Project (SolCAP). The SolCAP North American potato diversity panel is a compilation of elite potato germplasm from breeding programs across the U.S., as well as historical varieties from the NRSP-6 potato gene bank (Hirsch et al., 2013), and includes tetraploid species, diploid species, wild species and some diploid and tetraploid genetic stocks. For this study only the 190 cultivated tetraploid lines that contained both phenotypic and genotypic data were analyzed. Additional information about these lines was provided including release dates and the classification of each variety into one of six market classes: French Fry processing, Chip Processing, Table Russet, Round White table, Yellow and Pigmented (Hamilton et al., 2011). Genotyping was done with an Infinium SNP array of 8303 markers, and analyses to determine allelic dosages were performed with GenomeStudio. Poor quality SNPs, and SNPs unable to distinguish between the heterozygous classes were removed, leaving 3763 bi-allelic SNPs with reliable information on allelic dosages (Hirsch et al., 2013). For all calculations utilizing SNP information, the marker matrix was coded categorically (AAAA, AAAB, AABB, ABBB, and BBBB) or as a numerical measure of the number of alternate alleles present (0,1,2,3, and 4), where “A” is the reference allele and “B” the alternative allele.

Genomic Prediction was conducted for the three quantitative traits, especially important to the French fry and potato chip markets: tuber length (millimeters), tuber fructose and sucrose content (milligrams *gram*^−1^ fresh weight). Information on these traits were reported in the study by Rosyara et al. (2016), and were chosen so that for this study, we examine traits with high broad-sense heritabilities like tuber length and fructose content (*h*^2^ = 0.91 and *h*^2^ = 0.85, respectively), and sucrose content, a trait with intermediate heritability (*h*^2^ = 0.67) (Rosyara et al., 2016). These traits, among others were measured in as many as four environments (New York 2010, Wisconsin 2010, New York 2011, and Wisconsin 2011) however not all traits were measured in all environments. The trials consisted of a randomized complete block design with two replicates in each environment and using a linear model accounting for experimental design variables, phenotypic values were generated as the best linear unbiased estimator (BLUE) (Rosyara et al., 2016).

### Analyses

#### Population Structure

To assess population structure for the definition of strata, the marker data was analyzed using three methods: Principal Components Analysis (PCA), Discriminant Analysis of Principal Components (DAPC) and Analysis of Molecular Variance (AMOVA). In a population with distinct sub-divisions, a significant portion of the genetic variability of the population can be attributed to the differences between sub-populations. AMOVA estimates variance components of various factors, including the contribution of subgroups to a population's total variability (Excoffier et al., 1992). Population structure can also be visualized and quantified using Principal Components (Jombart, 2008). Market classes were given for the SolCAP North American diversity panel, and to visualize the extent of separation between these classes, DAPC was implemented. Unlike PCA which looks at overall variability (between and within classes), DAPC maximizes the between group variation with respect to the variation within groups (Jombart et al., 2010).

#### Sampling Methods

To evaluate training set construction methods, prediction accuracies were compared. Accuracy was defined as the correlation between observed phenotypic values and genotypic values of the validation/test set predicted by the corresponding genomic prediction model. The underlying hypothesis is that the prediction accuracy may be affected by the training set used to calibrate the model; a training set that does not cover the design space will result in poor predictions of the test set. In this study, four methods for constructing the training set were compared: simple random sampling, stratified random sampling, genetic distance sampling and the CDmean method.

- **Simple Random Sampling (SRS):** Training set construction is equivalent to taking a subset of a larger set. For simple random sampling, members of this subset are chosen randomly and completely by chance so that each individual from the panel has an equal probability to be selected for the training set.
- **Stratified Random Sampling (STRAT):** Using the population analysis results to define strata, this method randomly selects individuals from each sub-population, ensuring that every sub-population is represented in the sample, while maintaining the same strata proportions.
  $$n_{S}=\frac{n}{N}\times N_{S}$$
  For the above equation *n*_*S*_ is the number of individuals in the sample from stratum *S*, *N*_*S*_ is the number of individuals in the population from stratum *S*, while *n* and *N* are the total sample size and total number of individuals, respectively.
- **Genetic Distance Sampling (GD):** This method requires as input, the distances between genotypes calculated from the marker data. From the initial pool, one individual is randomly selected and all individuals within a radial distance *r* are discarded and will no longer be candidates for sampling. This ensures that the next individual sampled will not be genetically similar to the first individual. From the remaining set, a second individual is selected and again, all individuals within a genetic distance of *r* are discarded. This process is continued until the desired training set size is attained. The size of the sampling radius *r*, is dependent on the desired sample size. A larger sample size requires a smaller *r* and vice versa. The method is described in more detail in Jansen and van Hintum (2007), and is implemented in Genstat (VSN-International 2015). This implementation requires a similarity matrix, with a diagonal of 1′*s* and the off-diagonals in the range of [0, 1].
  This similarity matrix comprises of pairwise measures of genetic similarity between individuals, which Jansen and van Hintum calculated using the simple matching coefficient. The authors go on to suggest the Jaccard's similarity index as a suitable alternative (Jansen and van Hintum, 2007). Suggestions for calculating the genetic distance between polyploids have been made in literature (Dufresne et al., 2014), and include the Jaccard similarity index. As part of this study, four genetic distance measures were compared. These measures were chosen due to their suitability for SNP data, polyploids and their frequency of use.
  1. Nei's Genetic Distance makes the biological assumptions of an infinite alleles model and that genetic distances are a result of mutation and drift (Nei, 1972). A categorical marker matrix (AAAA, AAAB, AABB, ABBB, and BBBB) was used as input, and the Nei's distance between two individuals *X* and *Y* was calculated using the formula:
    $$D_{XY}=-ln\frac{\sum_{i=1}^{2}\sum_{j=1}^{r}p_{ij,x}p_{ij,y}}{\sqrt{\sum_{i=1}^{2}\left(\sum_{j=1}^{r}p_{ij,x}^{2}\right)\sum_{i=1}^{2}\left(\sum_{j=1}^{r}p_{ij,y}^{2}\right)}}$$
    where *r* represents the total number of markers and *p*_*ij,x*_, is the proportion of the *i*^*th*^ allele present at the *j*^*th*^ locus in individual *X*. For example, a particular locus with genotype AAAB has *p* = 0.75 for the reference allele “A.” This study uses bi-allelic markers hence the summation over the number of alleles is limited to two terms ($\overset{2}{\underset{i=1}{\sum }}$). The distance matrix was converted to a similarity matrix by subtracting from one, in accordance with the requirements of the genetic distance sampling algorithm.
  2. Euclidean Distance makes no biological assumptions as it is purely a geometric distance measure. Using the numerical coding of the marker matrix (0,1,2,3, and 4) this measure calculates the distance between two individuals *X* and *Y*:
    $$D_{XY}=\sqrt{\overset{r}{\underset{j=1}{\sum }}{\left(X_{j}-Y_{j}\right)}^{2}}$$
    In this equation *Y*_*j*_ can be interpreted as the number of alternate alleles at the *j*^*th*^ marker in individual *Y*. The Euclidean distance matrix was converted to the similarity measure, and scaled to fit within the desired range [0, 1] using the following transformation:
    $$1-\left(\frac{D_{XY}}{max\left(D_{XY}\right)}\right)$$
  3. Jaccard's Similarity Index does not make any biological assumptions and requires as input the numerical representation of the SNP data. The distance between two individuals *X* and *Y* is calculated as:
    $$D_{XY}=\frac{\sum_{j=1}^{r}|X_{j}\cap Y_{j}|}{\sum_{j=1}^{r}|X_{j}\cup Y_{j}|}$$
    In the above expression, |*X*_*j*_ ∩ *Y*_*j*_| is the number of alternate alleles common to both individuals *X* and *Y* at the *j*^*th*^ marker, while the term |*X*_*j*_ ∪ *Y*_*j*_| refers to the total number of alternate alleles at this same marker for individuals *X* and *Y*, without repetition (for tetraploids the maximum value for this term is 4). The resulting output was then converted to a similarity matrix.
  4. Kosman and Leonard's Genetic Distance differs from previously mentioned genetic distance measures as it takes into account the ploidy level of the individuals (Kosman and Leonard, 2005). With the numerical marker matrix of allele dosages (0, 1, 2, 3, and 4) as input, this measure calculates the similarity between two individuals *X* and *Y*:
    $$D_{XY}=\frac{1}{r}\overset{r}{\underset{j=1}{\sum }}\frac{X_{j}\cap Y_{j}}{q}$$
    In this equation, *X*_*j*_ ∩ *Y*_*j*_ corresponds to the number of shared alleles at the *j*^*th*^ marker, which is divided by *q* the number of chromosome copies (4 for tetraploid), and averaged over all *r* markers.
- **Generalized coefficient of determination (CDmean):** The generalized coefficient of determination is a training set selection method based on the maximization of the precision of the prediction of differences (or contrast) between the average value of the entire population of candidate individuals and each individual in the test set (Rincent et al., 2012). Maximizing Equation 1 (below), leads to the maximization of the precision of contrasts.
  (1) $$\begin{array}{l} CD\left(c\right)=diag\left[\frac{c^{\prime }\left(A-\lambda {\left(Z^{\prime }MZ+\lambda A^{-1}\right)}^{-1}\right)c}{c^{\prime }Ac}\right] \end{array}$$
  Where *c* is the matrix of contrasts between each individual without phenotype information and the average of the candidate individuals, λ is the ratio between the residual and additive genetic variances, *Z* is a design matrix that will be used in GP models to relate observations to genomic values (seen in Equation 3 in a later section), and *M* is an orthogonal projector on the subspace spanned by the columns of the fixed effects design matrix, *X* (also seen in Equation 3), such that *M* = *I* − *X*(*X*′*X*)^−^*X*′. *A* is the additive realized genomic relationship matrix as calculated by VanRaden (2008):
  (2) $$\begin{array}{l} A=\frac{QQ^{\prime }}{2\sum_{j=1}^{r}p_{j}\left(1-p_{j}\right)} \end{array}$$
  Where *Q* is a matrix calculated from *Q*_*ij*_ = *W*_*ij*_ + 1 − 2*p*_*j*_, with *i* individuals (rows) and *j* markers (columns). The term *p*_*j*_ is the frequency of the reference allele of the *j*^*th*^ marker and *W* is the numerical marker matrix, centered and scaled such that genotypes coded as allele dosages {0, 1, 2, 3, 4} now become {−1, −0.5, 0, 0.5, 1}. The supporting literature (Rincent et al., 2012) reports negligible differences in selected samples, when different estimations of the genomic relationship matrix are used. This was confirmed in a small preliminary analysis where three different methods of calculating this matrix were tested, as prediction accuracies were similar between methods. Therefore, the VanRaden method was chosen as it is well-known in the context of genomic prediction.
  From the description of λ above, its calculation requires an estimate of trait heritability (*h*^2^) and though we have phenotypic data and can therefore estimate this value for the traits in question, this may not always be the case in practice. Often the decision of which genotypes are to be put in the field to garner phenotypic measurements, is made before estimates of heritability can be performed, as this calculation requires phenotypic data. Secondly, the individuals to be selected may not have to be chosen on the merit of one single trait, but rather by more traits with varying degrees of heritability. The supporting literature (Rincent et al., 2012), suggests and provides evidence that the use of an intermediate value of heritability (example 0.5), selects training sets very similar to those using more extreme values of heritability. A small preliminary analysis was performed and these results confirmed that the heritability estimate had little to no impact on prediction accuracy and therefore, for this study, the heritability input for the CDmean method was set at 0.5 for all traits.
  The code for implementing both the CDmean method and genetic distance sampler, can be found in the Supplementary Material.

#### Prediction Scenarios

**Figure 1:**
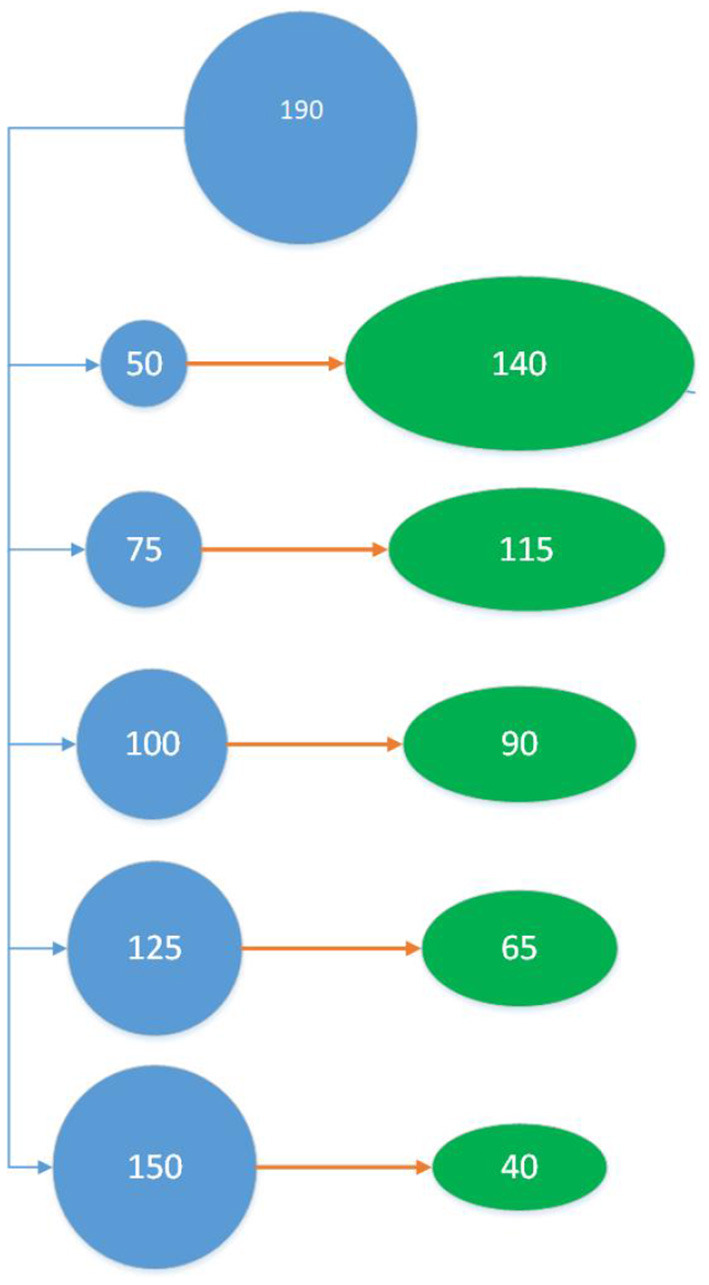
Training-Validation (TV) Scheme: Out of 190 individuals, 50 individuals are sampled as the training set to train the model and validated using the remaining 140 individuals in the green oval. This is repeated 100 times for each training set sampling method. The entire process was then repeated for training set sizes 75, 100, 125, and 150 (which impacts the size of the validation set).

The training set selection methods were compared by two cross validation schemes: the training-validation (TV) scheme and the training-test (TT) scheme. The TV scheme follows a typical cross-validation approach where a portion of the individuals are used to train the model (training set) and those not part of the training set, used to evaluate model prediction accuracy (validation set). The effect of training set size was assessed by choosing 50, 75, 100, 125, and 150 individuals out of the total 190 with each sample size repeated 100 times. We must consider that the training and validation sets are complementary, therefore the size of the validation set depends on the size of the training set, so comparisons across training set sizes are not equally precise (see Figure 1). Additionally, when a diverse set of individuals are chosen, an equally diverse set of individuals are left behind, which may impose some bias. Another important consideration from an application point of view, is that in a real situation a breeder will have individuals that were not phenotyped at all, so we want to assess the performance of the sampling methods assuming that the information of some of the individuals is truly absent, which the TV scheme does not fully represent.

**Figure 2:**
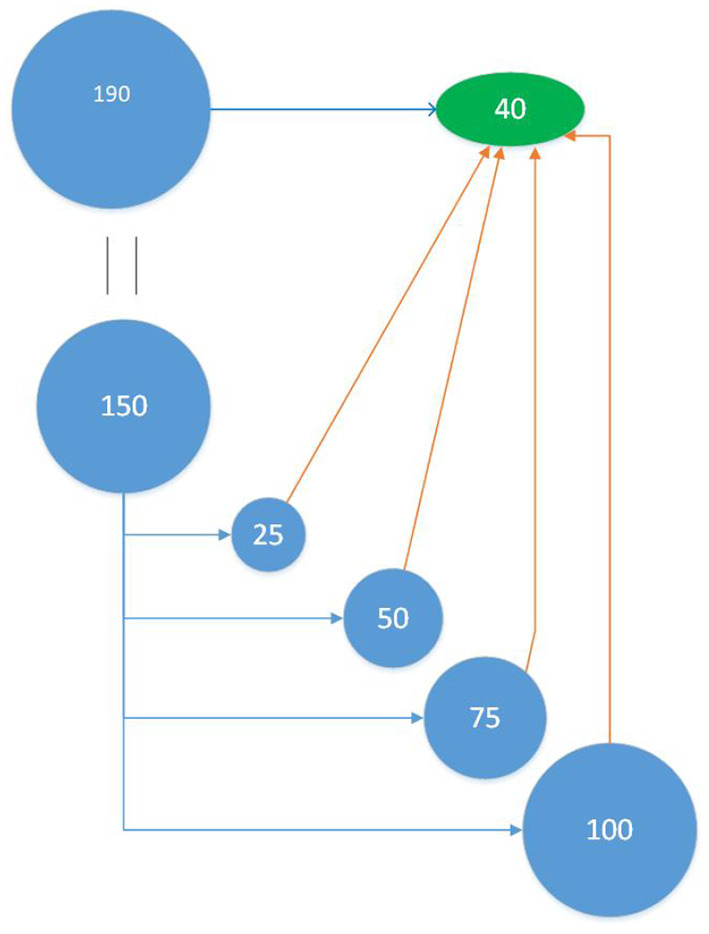
Training-Test (TT) Scheme: Out of the 190 individuals a test set of 40 individuals were randomly selected. The remaining 150 individuals were now candidates for the training set and from this set, 25 individuals were selected and used to train the model that predicts the test set. This was performed 50 times for each training set sampling method. This process was repeated for 50 different test sets. The analysis was performed at varying training set sizes (25, 50, 75, and 100).

Therefore, a second approach (TT scheme) was used where the composition and size of the validation (test) set, is independent of the composition and size of the training set. In each realization of the TT scheme, we first randomly sampled 40 individuals as test set leaving the remaining 150 as the pool from which to sample the training set. Following the different sampling methods, we chose 25, 50, 75, and 100 genotypes from the remaining 150, as training set (see Figure 2). In turn, the sampling of the training set was repeated 50 times, making the accuracy of a particular realization the average of 50 repetitions of the same sampling method, that sample a certain number of individuals to train a particular statistical model and predict a given test set. This entire process was then repeated 50 times, each time with a new test set. This methodology ensures that all training set selection methods train a model that predicts the same test set and gives better assessment of training set selection methods. In addition, we investigated larger sizes of the test set (70, 95). For a test set of 70 individuals, training set sizes are the same as seen above (25, 50, 75, and 100), but for a test set of 95 individuals, the training sets evaluated were of sizes 30, 45, 60, and 75.

#### Genomic Prediction Models

The purpose of this study was to uncover a superior training set sampling method based on the accuracy of predictions. These predictions were generated with three different whole genome regression models, in order to investigate the presence/absence of an interaction between training set selection method and genomic prediction model.

- **GBLUP:**
  (3) $$\begin{array}{l} y=X\beta +Zu+\epsilon \end{array}$$
  For Equation 3, *y* is a vector of phenotypic BLUEs, β is a vector of fixed effects (only the intercept in our case), *u* is a vector of genotypic values with distribution $u\sim N\left(0,A\sigma_{g}^{2}\right)$. *A* is the genomic relationship matrix as calculated in Equation 2 and $\sigma_{g}^{2}$ is the additive genetic variance. *X* and *Z* are design matrices as described previously and ϵ is the vector of residuals with distribution $\epsilon \sim N\left(0,\sigma_{\epsilon }^{2}\right)$. $\sigma_{\epsilon }^{2}$ is the residual variance.
- **RKHS:** The model for Reproducing Kernel-Hilbert Spaces (RKHS) is the same as Equation 3, with one difference in that the genotypic values have a different distribution: $u\sim N\left(0,K\sigma_{g}^{2}\right)$. The genomic relationship matrix *A*, is replaced by the kernel matrix, $K=exp^{-\frac{D}{\theta }}$, where D is a Euclidean distance matrix and θ a tuning parameter. The tuning parameter controls how fast the relationship between two genotypes decays as the distance between the corresponding pairs of marker vectors increases (Jiang and Reif, 2015). To estimate θ, a grid search was conducted between (0, 1] and the value that gave the maximum log-likelihood was chosen (Endelman, 2011). Applying RKHS in this study allows for the implicit modeling of non-additive effects.
- **BAYES Cπ:**
  (4) $$\begin{array}{l} y=X\beta +Wb+\epsilon \end{array}$$
  In Equation 4, where *W* is our matrix of marker information, *b* is a vector of marker effects. Bayes Cπ assumes that marker effects come from a mixture distribution with a proportion of markers (π) having zero effect and the remainder (1 − π) having non-zero effects, such that for the *j*^*th*^ marker:
  $$b_{j}=\{\begin{array}{ll} 0 & :\text{ with probability }\pi \\ \sim N\left(0,\sigma_{b}^{2}\right) & :\text{ with probability }1-\pi \end{array}$$
  The proportion of zero effect markers π, was estimated from the data. For this study, 5,000 iterations were performed with 2,500 discarded as burn-in, with the BGLR package (Prez and Campos, 2014). In preliminary analyses, larger number of iterations were tested and the outcomes were identical, in terms of prediction accuracy and convergence diagnostics.

#### Prediction Accuracy

As mentioned in previous sections, the ranking of the training set construction methods will be based on a measure of prediction accuracy. For both the TV and TT schemes, the observed phenotypic values of the training set are fed to the statistical models to estimate marker effects, while the phenotypic values of the validation (TV scheme) and the test set (TT scheme), are hidden from the model. Predictions are made on those individuals with hidden phenotypes, and the prediction accuracy is defined as the Pearson correlation between observed phenotypic values and the predicted genotypic values. Factors that may influence prediction accuracy are sample size, statistical model and the training set construction method, as well as various interactions between these factors. To answer this question, an Analysis of Variance (ANOVA) was carried out where the correlation (prediction accuracy) is treated as the response variable such that *accuracy* = *f*(*size, model, method*) in a full factorial model. To conform to normality assumptions, these correlations (accuracies) were transformed using Fisher's *z* transformation, $z=\frac{1}{2}\left(ln\left(\frac{1+r}{1-r}\right)\right)$.

All analyses were executed in R Core Team (2020), except for genetic distance sampling which was performed in Genstat as mentioned previously.

## Results

The 3,763 SNPs were reduced to 3,262 after the following filtering steps. For the 190 phenotyped tetraploid lines, monomorphic markers, unmapped markers, markers with a minor allele frequency of <5% and markers with missing values for more than 30 of the 190 individuals were removed.

### Population Structure

The classification of the population into the six market classes, gives two subpopulations with <20 individuals. This is not ideal for stratified sampling as parameter estimates from these very small subgroups will produce large standard errors. Furthermore, based on past population structure results for this diversity panel, there are indications that some of these sub-populations can be merged.

**Figure 3:**
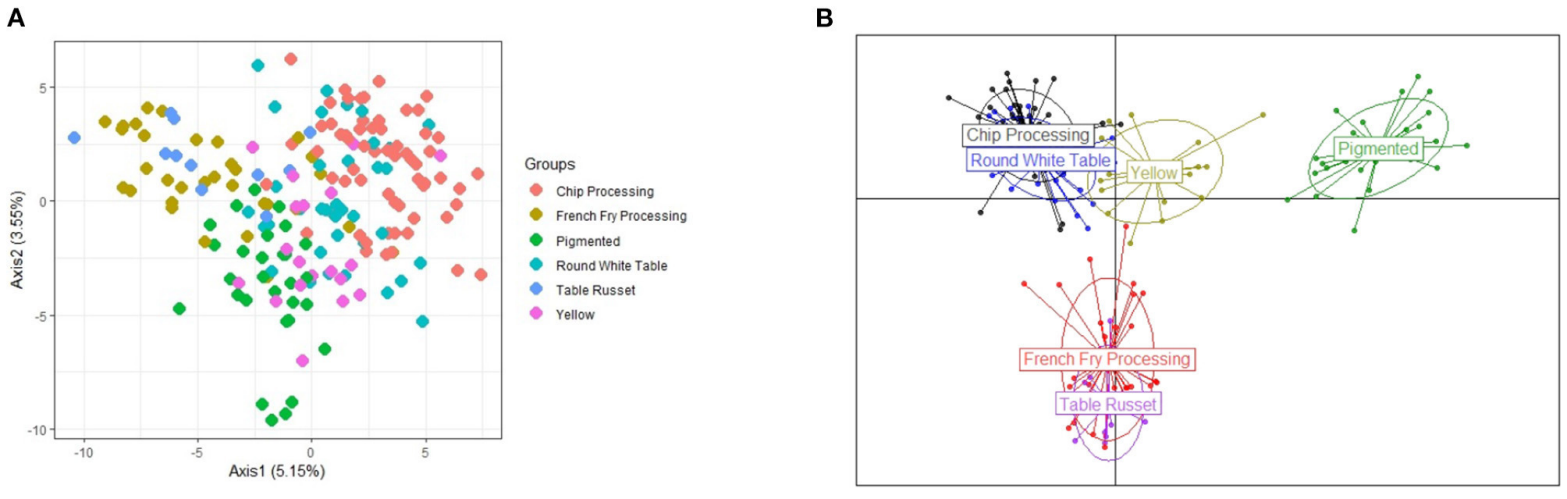
Illustration of the population structure explained by the first 2 principal components (PCA) and discriminant analysis of the principal components (DAPC) showing the separation of the 6 market classes. **(A)** PCA with market classes. **(B)** DAPC of market classes.

PCA and DAPC results show that the six market classes can indeed be reduced to a smaller number of groups (Figure 3). Principal Components Analysis (Figure 3A) found that the first two principal components account for <10% of the explained variance with the 1st principal component capturing 5% of the variability, while the 2nd component explains only 3.55%. The decision on which classes should be merged were made by inspecting the results from DAPC (see Figure 3B). For this analysis, 100 principal components and three discriminant functions were chosen. From here we see that the French Fry processing and Table Russet market classes show considerable overlap, as well as the Chip processing and Round White table market classes.

The pigmented class is clearly separated but one question arose: Where does the yellow market class belong? AMOVA analyses found that genetic variation due to population structure was the highest (16.6%), when the yellow class was placed with chip processing and round white table classes, as suggested by the DAPC plot (Figure 3B). Other population structure configurations were analyzed, including each of the six separate market classes as its own sub-population, as well as maintaining the three clearly separated groups seen in Figure 3B, but placing the yellow market group with the pigmented class (see Appendix). Placing the yellow class with the chip and round-white class, instead of the pigmented class was supported by both AMOVA analyses and pairwise Fst statistics between the groups. Between Yellow and Pigmented, *Fst* = 0.0165, while between Yellow and Chip Processing-Round White table, *Fst* = 0.0098 (where Fst values closer to zero indicate populations that are more genetically similar). For the remainder of the study, the discrete population structure used for stratification is defined by the three groups suggested in Figure 3B, with the yellow market class merged with the neighboring group of chip processing and round white table potatoes.

### Genetic Distance Measures

Four different genetic distance measures were used to perform genetic distance sampling, and the sampled individuals were used to train the model. Prediction was performed on the left out individuals as described in the TV scheme. The similarities (correlations) between the different genetic distance matrices were assessed by a Mantel test (Table 1).

**Table 1:** Correlation between different genetic distance matrices.

	**Euclidean**	**Nei**	**Jaccard**
Nei	0.989		
Jaccard	0.927	0.941	
Kos.&Leo.	0.967	0.975	0.977

There is very little difference between the distance measures for the material in this study. The lowest correlations (0.927 and 0.941) occurred with the Jaccard distance measure, however this degree of similarity is still quite high.

**Figure 4:**
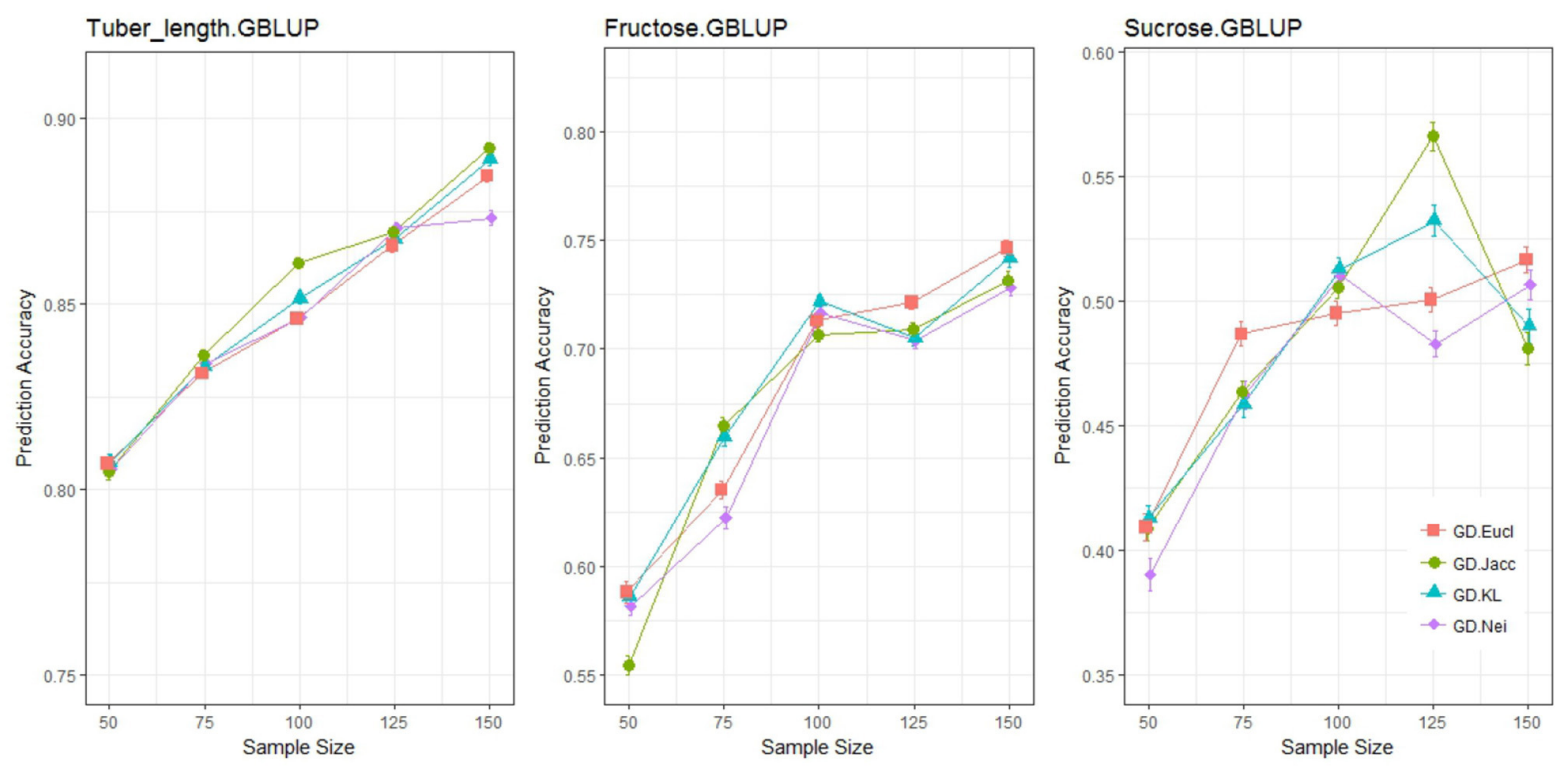
Prediction accuracy under different distance measures for genetic distance sampling with a GBLUP statistical model. Each graphical window represents a different trait, with varying sample sizes on the *x*-axis and the outcome variable, prediction accuracy on the *y*-axis. The different colored lines represent different distance measures.

The prediction accuracies from a common GBLUP model were quantified for three different traits (tuber length, fructose and glucose content), at sample sizes ranging from 50 to 150, for different genetic distance measures (Figure 4). It can be concluded that the choice of distance measure had a minor impact on prediction accuracy. Prediction accuracy is expected to increase as sample size increases and Euclidean distance was the most consistent measure across all traits. The remaining three measures displayed non-monotonically increasing prediction accuracies as sample size increased. Additionally, the Kosman Leonard distance, along with having very little application in literature, becomes computationally heavy when there are more than 10,000 markers. For this study, the Euclidean distance will be used henceforth when applying genetic distance sampling for training set construction.

### Genomic Prediction: TV Scheme

**Figure 5:**
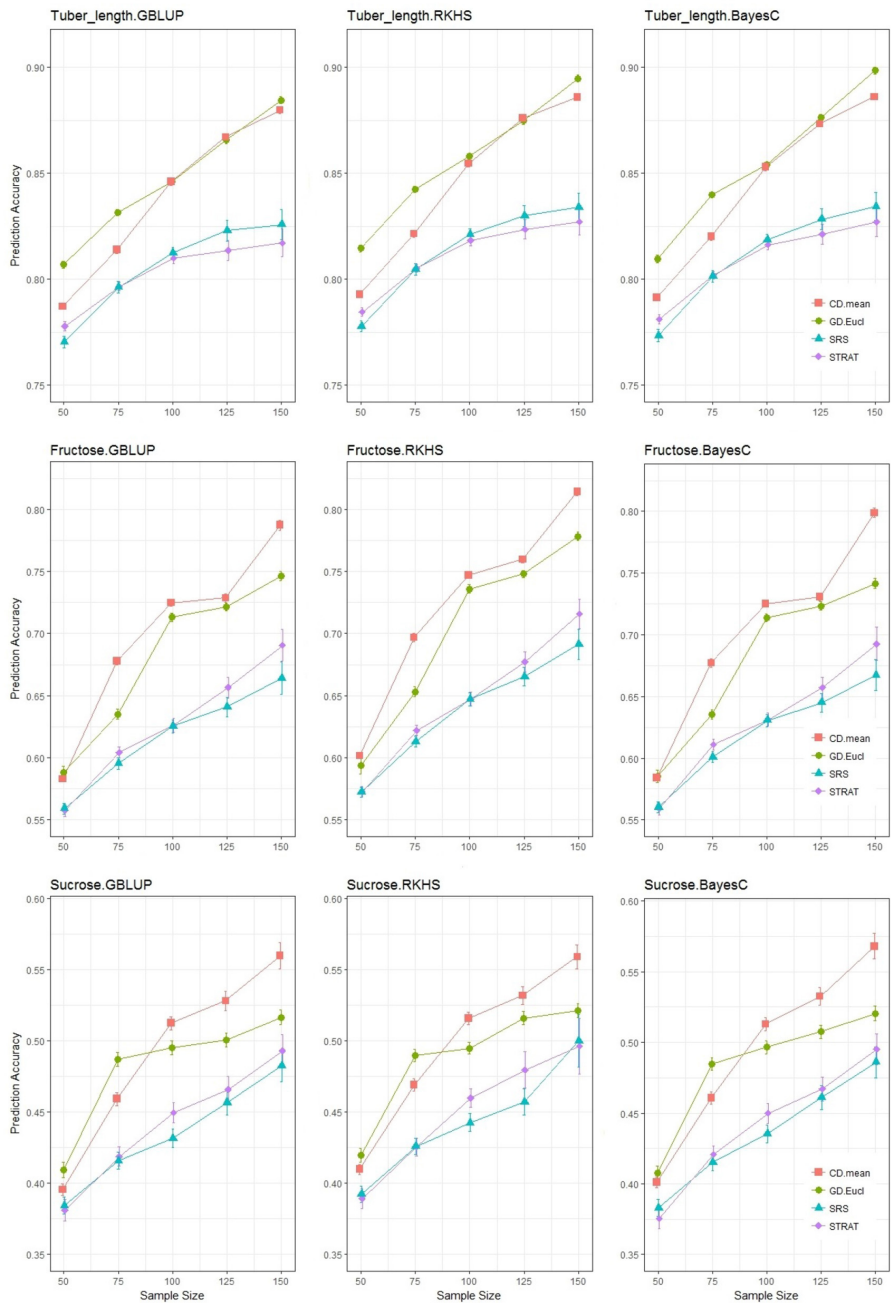
Prediction accuracy for the 3 traits under the TV scheme (training and validation only). Each graphical window displays a different trait-statistical model combination, with varying sample sizes on the *x*-axis and prediction accuracy on the *y*-axis. The different colored lines represent different training set selection methods.

After determining a suitable distance measure for genetic distance sampling, methods for acquiring the training set were compared (Figure 5).

Each row of Figure 5 shows a single trait with the different genomic prediction models, and compares the prediction accuracies across sample sizes ranging from 50 to 150. For all traits, a difference is clearly observed between training set selection methods: with simple random and stratified random sampling (random methods) behaving similarly while genetic distance sampling and the CDmean method (analytical methods) sampled training sets, gave more accurate predictions. As expected, an increase in sample size increased prediction accuracy, but this was at a higher rate when using the analytical methods of selecting individuals. The lines above and below the points indicate the standard errors of the estimate of average accuracy, and the random sampling methods resulted in larger standard errors than the analytical methods. For all trait-statistical model combinations, the random methods of selecting the training set were not significantly different; stratifying the population before sampling, did not improve the accuracy of genomic prediction, in comparison to a simple random sample of the training set. Even though the analytical methods consistently performed better than the random methods, the comparative performance between the two analytical methods varied with traits. For tuber length, the genetic distance sampler selected a more optimal training than the CD method at lower sample sizes (50 and 75), but this difference diminished as the size of the training set increased. The CD mean method generally outperformed the genetic distance sampler in predicting fructose and sucrose, more noticeably so at higher sample sizes. Interestingly, at sample size 50 and 75, genetic distance sampling led to more accurate predictions of sucrose content, a result also observed for tuber length. Despite these minor differences, the results across all traits give clear support for utilizing analytical methods of selecting the training set, and some indication that the CDmean method is the better of the two analytical training set selection strategies.

The results shown in Figure 5, include information about the three different statistical models. The possibility of an interaction between statistical model and training set selection method was evaluated in this study, and results from an ANOVA analysis were used to quantify the impact of this interaction (Table 2).

**Table 2:** ANOVA table showing the significance of the statistical model, sample size, training set selection method, and interactions for the prediction accuracies of tuber length (TV Scheme).

	**df**	**SS**	**MS**	***F*-value**	**Pr(>F)**
Method	3	0.278	0.0925	319	< 2 × 10^−16^
Sample size	1	0.386	0.386	1,330	< 2 × 10^−16^
Model	2	9.08 × 10^−3^	4.54 × 10^−3^	15.7	1.27 × 10^−5^
Method: sample size	3	0.0605	0.0202	69.6	4.66 × 10^−15^
Method: model	6	5.58 × 10^−4^	9.30 × 10^−5^	0.321	0.922
Sample size: Model	2	9.42 × 10^−4^	4.71 × 10^−4^	1.63	0.211
Method: sample size: model	6	3.53 × 10^−4^	5.88 × 10^−5^	0.203	0.974
Residuals	36	0.104	2.90 × 10^−4^		

The magnitude of the *F*-values in Table 2 indicate how important a term is for predicting the outcome, which is the accuracy of genomic predictions in this case. The most important factor for driving genomic prediction accuracies is sample size, followed by the training set selection method and then the interaction between these two variables. The interaction of interest, between sampling method and statistical model, explains very little of the variation in prediction accuracy. There is no particular combination of sampling method and statistical model that results in more accurate predictions but rather, the main effects of these two variables. Results in Table 2 are based on tuber length, and these results were consistent across all traits, with sampling method being highly significant, and its interaction with statistical model, non-significant. An interesting result is the significant interaction between sample size and sampling method which was consistent across all traits. This means that the sampling methods do not benefit equally from an increase in sample size, a result also observable from Figure 5.

For fructose, when the sample size is tripled (from 50 to 150), simple random sampling and stratified sampling improved by 19 and 23%, respectively, whereas genetic distance sampling and the CDmean method resulted in improvements of 27 and 31%, respectively. For sucrose, the CDmean method showed a 37% improvement by tripling the sampling size while simple random sampling improved by 25%. The relative improvement of using a analytical sampling method was greater for sucrose and fructose content. At the median sample size of 100, CDmean showed an improvement in prediction accuracy of 4, 14, and 13% for tuber length, fructose and sucrose content, respectively, when compared to simple random sampling. The genetic distance sampler for these traits (tuber length, fructose and sucrose content, respectively), showed improvements of 5.5, 10.5, and 10.5% in comparison to simple random sampling.

### Genomic Prediction: TT Scheme

As discussed before, the objectives for using Genomic Prediction may vary. In many cases the objective is to predict new breeding lines (or clones) and for this scenario we have randomly selected a test set of 40 out of the 190 individuals. These 40 individuals represent the independent test set, and all sampling methods will construct the training set from the remainder of individuals. The trained model then performs predictions for the test set. In this way, each sampling method predicts the same test set.

**Figure 6:**
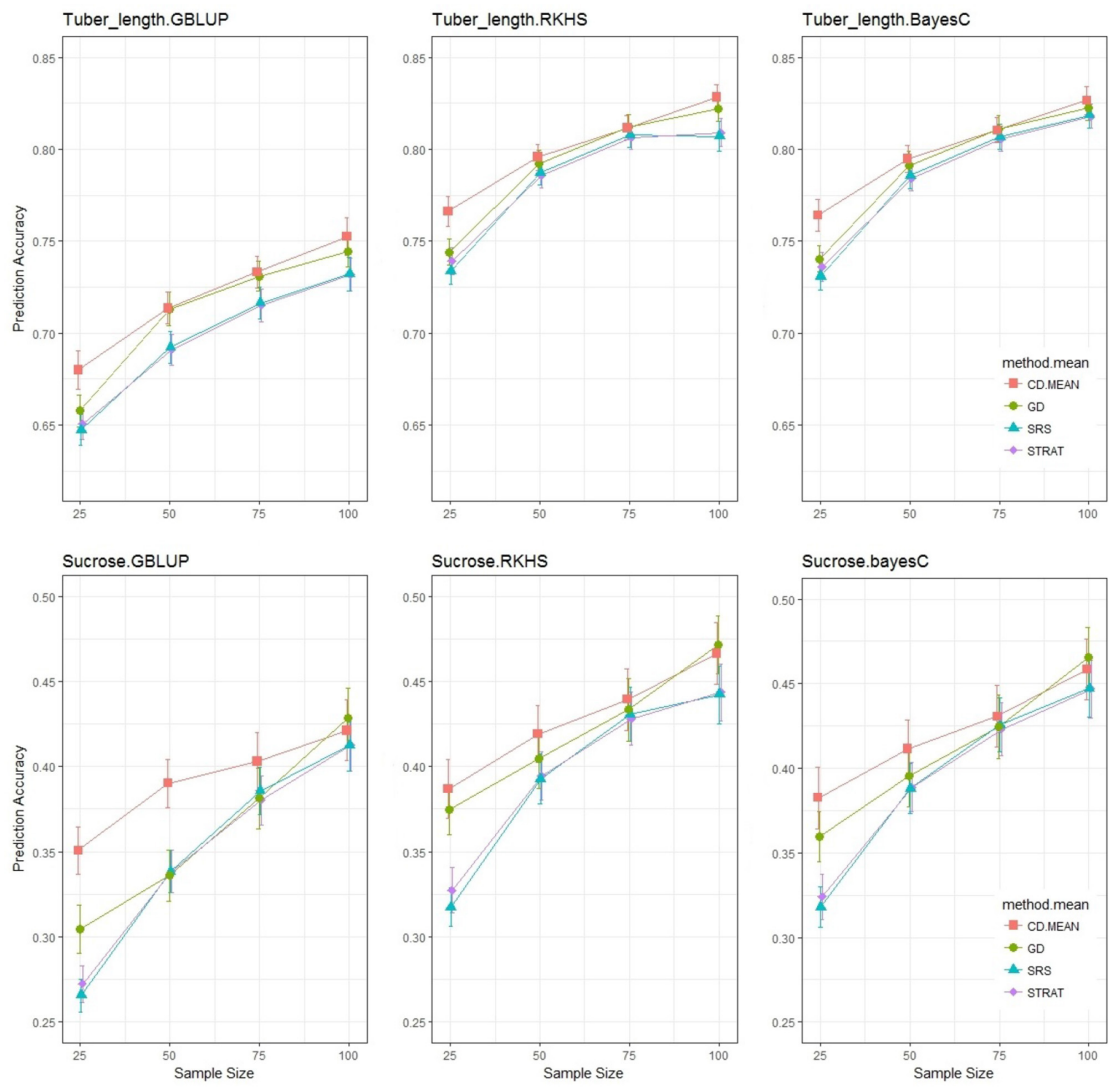
Prediction Accuracy for tuber length and sucrose content with a test set of size 40. Each graphical window represents a different trait-statistical model combination, with varying sample sizes on the *x*-axis and prediction accuracy on the *y*-axis. The different colored lines represent different training set sampling methods.

Similar to the previous section, we looked at the prediction accuracy for three statistical models with sample sizes ranging from 25 to 100, and compared the impact of the sampling method (Figure 6). The accuracies of the TT Scheme are a bit lower and are accompanied by larger standard errors than those observed in the TV Scheme, due to the application involving a test set, which is usually more difficult to predict but represents a more realistic scenario encountered by breeders. Nonetheless the decrease in accuracy was not drastic. The differences between sampling methods is still present, but less obvious than in the TV Scheme, especially at higher sample sizes where the accuracies of the various sampling methods converged as was expected, due to the significant overlap of individuals sampled in a limited population space of 150 varieties. This convergence is not observed in the TV Scheme and will be discussed in another section. At the lower sample sizes, where the potential overlap of training sets is reduced, the analytical methods give significantly higher accuracies than the random methods. For tuber length, genetic distance sampling and the CDmean method result in similar prediction accuracies for sample sizes ≥50, but for sucrose content, this similarity was dependent on the statistical model applied.

In comparison to the TV scheme, the results of the TT scheme exhibit a more significant impact due to statistical model, and to test whether there is an interaction with the sampling method an ANOVA analysis was conducted.

Similar to the results from the TV scheme shown in Tables 2, 3 shows that for the TT scheme, sample size was the most important factor driving prediction accuracy, and there was no interaction between the statistical model and the sampling method. It was noteworthy that the hierarchy of importance of predictive variables was quite different between schemes. Our factor of interest, sampling method, though still significant in the TT application, was not the second most important variable as seen before, but replaced by statistical model in the hierarchy. Also different to the TV scheme, the TT scheme results show no interaction between sample size and the sampling method. The results in Figure 6 and Table 3 were similar to those observed for fructose content, with CDmean only slightly outperforming the rest, but with even less differentiation between sampling methods. The ANOVA analysis for fructose content (not shown), showed that there was little to no impact of different training set construction methods.

**Table 3:** ANOVA table showing the importance of the statistical model, sample size and training set selection method and interactions for the prediction accuracies of tuber length (TT Scheme).

	**df**	**SS**	**MS**	***F*-value**	**Pr(>F)**
Method	3	0.0136	4.52 × 10^−3^	5.94	0.00352
Sample size	1	0.213	0.213	280	9.75 × 10^−15^
Model	2	0.404	0.202	265	< 2 × 10^−16^
Method: sample size	3	8.79 × 10^−4^	2.93 × 10^−4^	0.385	0.765
Method: Model	6	3.71 × 10^−4^	6.18 × 10^−5^	0.0810	0.998
Sample size: Model	2	2.01 × 10^−3^	1.01 × 10^−3^	1.32	0.285
Method:sample size:model	6	3.45 × 10^−4^	5.75 × 10^−5^	0.0760	0.998
Residuals	24	0.0183	7.61 × 10^−4^		

*Higher F-values or Mean Sum Sq values indicate higher predictive power of a variable*.

Although this paper does not primarily focus on statistical models, it is still interesting to observe the differences in predictive performance (Table 4). For all traits, the GBLUP model gave the lowest accuracy of predictions, while the Bayes C model worked just as well as the RKHS model.

**Table 4:** Marginal means and standard errors for prediction accuracy for varying combinations of statistical model (columns) and trait (rows).

	**GBLUP**	**RKHS**	**BAYES.C**
Tuber length (s.e. = 0.010)	0.708	0.792	0.792
Fructose (s.e. = 0.007)	0.450	0.580	0.571
Sucrose (s.e. = 0.005)	0.364	0.412	0.406

Application of the TT scheme to breeding programs, usually involves a test set of hundreds or even thousands of new potential cultivars. In this study it was impossible to emulate this application, still the impact of increasing the test size was investigated. For this investigation, we conducted the same analyses as seen in TT scheme but used a larger test set (70 and 90 individuals). There were no changes in the findings; the analytical methods, especially CDmean, sampled training sets that predicted the test sets with greater accuracy than the random methods (results not shown). Similar to the results seen above, these differences disappeared at larger sample sizes and were only evident at smaller training set sizes, where the overlap of sampled individuals between methods was minimal.

## Discussion

Training set construction has been proven to be important for GP in diploids and in this study, shown to be important for GP in tetraploids. Both ploidy levels benefit from incorporating genomic information into analytical methods of sampling the training set, when compared to random methods that do not directly utilize genomic information.

Only 190 varieties were included in this study which may limit the extrapolation of results to traditional breeding programs. Breeders often make selections within a particular market group. In these scenarios, one must decide if to train models using only individuals belonging to the target market group or allow for the borrowing of information from other market classes. Our study was too small to answer this question, however it has been shown that combining individuals from both within and across market classes, can lead to predictions that are as good as, and often better than predictions made from exclusively within the market class (Rio et al., 2019). This is especially valid when the population structure is less definitive, as seen in this study.

As we are predicting heterogeneous populations, the use of interaction models may be considered (Lehermeier et al., 2015), where population structure induces heterogeneity of marker effects. For the interaction models, sub-populations should be large enough and definitive enough to estimate marker effects, but in this study our sub-populations were small. As population structure and size increase in magnitude, the Sparse Selection Index is another promising alternative (Lopez-Cruz and Campos, 2021).

### TV Scheme

For the training-validation scenario, results show a clear differentiation between the random methods (simple random sampling and stratified random sampling) and the analytical methods (genetic distance sampling and CDmean method). This separation between methods was not dependent on the statistical model used to make predictions which was confirmed by ANOVA analyses of prediction accuracies. As sample size increased so did prediction accuracy due to the fact that the estimation of marker effects is improved as the size of the training set increases, a finding also reported in studies of diploid crops (Rincent et al., 2012; Daetwyler et al., 2013; Bustos-Korts et al., 2016; Akdemir and Isidro-Sanchez, 2019). The improvement in accuracy awarded from a larger sample, is greater when applying an analytical method of sampling the training set. This result was supported by the significant interaction between sampling method and sample size. In training set construction for the TV scheme, we are essentially choosing a subset of individuals (randomly or analytically), that would calibrate the model used to make predictions on the subset of individuals not chosen for training; in essence the training set and validation sets are complements of each other. If we were to picture the population space spread evenly over four quadrants, and during training set construction, by chance all the members of a given quadrant belonged to the training set, then this quadrant would not be represented in the validation set. Our model would be trained in a space where it is not making predictions, leading to poor predictive potential. As the size of the training set increases using random methods, there is a chance that we continue to calibrate the model using redundant misrepresentative information, and the gain from increasing sample size is contested by predicting individuals that are genetically distant from the members of the training set. For this reason, the predictive power gained by adding one individual to the training set, is greater when using an analytical method for selecting the training set over a random sampling method. Analytical methods of training set construction allow the space occupied by the training set to be similar to that of the validation set, and as we increase the size of the training set, the information provided for model calibration continues to describe the entire genetic space in more detail, and not randomly over-represent a few areas with redundant information.

Taking a closer look at the random methods, we see that stratifying our samples had very little impact on prediction accuracy in comparison to simple random sampling. Diploid studies have shown that stratification based on population structure information may not be beneficial to constructing the training set, when there is no extensive separation between sub-populations (Isidro et al., 2015; Bustos-Korts et al., 2016). The panel of tetraploid potatoes used in this study showed little population structure, with only 16% of the total variation due to population structure. Therefore, stratification before sampling did not improve the accuracy of GP in comparison to simple random samples, similar to the results of comparable studies of diploid species with little sub-population separation (Isidro et al., 2015).

For sucrose and fructose content, the CDmean method sampled training sets that lead to more accurate predictions, however for tuber length, the genetic distance sampler chose an equally optimal training set. The extra information that is incorporated by the CDmean method, may help in choosing a training set, better equipped for traits that are harder to predict. In a study comparing training set construction methods among various diploid species and different traits (Bustos-Korts et al., 2016), the results showed no significant difference between the CDmean method and genetic distance sampler. Genetic distance sampling establishes a radius that is used to exclude individuals that are genetically close to a previously chosen member of the training set, and only considers genomic information (genetic distance). The CDmean method though, makes use of more information than the genetic distance sampler: trait variability and heritability. For traits that are influenced by non-genetic (environmental) factors, like fructose and sucrose content (Kumar et al., 2004), genomic information alone will not be as beneficial as having both genomic and phenotypic information. The combined information of trait variability and heritability, as well as genomic relationships between individuals, allows the CDmean method to construct a training set that produces higher accuracies for these traits. However, this necessity for phenotypic input information, in addition to the increased computational load, can make the CDmean method less attractive than genetic distance sampling.

#### Distance Measures

The differences between distance measures is very small when compared by correlation diagnostics. We were not able to explain the unexpected behavior exhibited by the Nei's, Jaccard and Kosman and Leonard genetic distances, where for fructose and sucrose content, the accuracy of predictions did not monotonically increase as sample size increased. The fact that Euclidean distance produced accuracies that were monotonically increasing with sample size, motivates the use of this measure in this study. However, this finding is not conclusive for all tetraploid studies: only bi-allelic markers were available for this study, but tetraploid individuals can have up to four alleles (Silva et al., 2005; Salimi et al., 2016). The Kosman and Leonard distances can utilize this information as it considers the number of different alleles at a given marker, and this is expected to produce better measures of distance between individuals (Kosman and Leonard, 2005; Dufresne et al., 2014), whereas the Euclidean distance uses a count of one particular allele (reference allele) as input to calculate genetic distances. This study did not contain the multi-allelic marker information needed to truly test the differences between the distance measures, and for scenarios like this that are limited to bi-allelic markers, the difference between distance measures will not be relevant.

### TT Scheme

To investigate the impact that the training set has on the prediction of new potential cultivars, the TT scheme was introduced which includes a randomly chosen test set. As expected, there was a decrease in overall prediction accuracy (Akdemir and Isidro-Sanchez, 2019). The divergence in accuracy between the random and analytical methods as sample size increased, observed in the TV scheme was not seen in the TT scenario. This is due to the fact that all methods predict the same group of individuals, and leave a limited pool of candidates to be selected for training the model. As a result, there was overlap in the training sets sampled by the various sampling methods. Secondly, the composition of the trainng set had no affect on the individuals where predictions were made, an unavoidable situation with the TV scheme. The TT Scheme reveals that the differences between training set construction methods depend on the scenario for which these methods are applied; scenarios with an independent test set (new breeding material) or instances where it may be more cost and time efficient to phenotype a few individuals and predict the rest (phenotyping platforms, TV scheme). These results are not conclusive, due to the moderate number of individuals in this study. The performance at the smaller sample sizes for the TT scheme may give an impression of what an ideal situation would look like, where there is a large population thus minimizing the overlap of individuals in the training sets constructed by the different methods. At these low sample sizes, the CDmean method constructed training sets led to more accurate predictions. Similar to the TV scenario, there is evidence that the utilization of both genomic and phenotypic information by the CDmean method is more beneficial for predicting traits highly influenced by non-genetic (environmental) factors. The genetic distance sampler maintains its position as the second best sampler. In spite of the limitation created by the population size, the evidence is still substantial: for GP of tetraploids in a training-test scenario, analytical methods of sampling the training set lead to better predictions, as seen also in diploids (Bustos-Korts et al., 2016; Akdemir and Isidro-Sanchez, 2019).

### Prediction Models

The performance of the prediction models can be explained by the architecture of the traits analyzed. GBLUP models work best for traits controlled by many small effects while models that perform marker selection are better suited for traits that are controlled by a few large effect QTL (de los Campos et al., 2013). A previous Genome Wide Association Study (GWAS) was conducted on the same diversity panel as this study, where significant QTLs were detected for tuber length, but not for sucrose and fructose content (Rosyara et al., 2016). Other studies have found that sucrose and fructose content are controlled by a small number of loci (Bradshaw et al., 2008; Sliwka et al., 2016; Rak et al., 2017). It is therefore not surprising that the BayesCπ model was able to make better predictions of all three traits in comparison to the GBLUP model.

Having four copies of each chromosome, one may expect that tetraploids exhibit more inter-locus interactions (epistasis) in comparison to diploids (Stich and Gebhardt, 2011). When non-additive effects like dominance and epistasis are present, they can be captured with the RKHS model (Gianola and van Kaam, 2008). Tuber length did not benefit from accounting for these effects while sucrose and fructose content showed little improvement. Fry color, strongly related to sugar content (Pritchard and Adam, 1994), can attribute the majority of its variability to additive effects, however there is a small contribution by non-additive effects (Endelman et al., 2018). This helps to explain the small but present improvement of the RKHS model over the BayesCπ model for these two traits.

## Conclusions

- Genomic prediction of individuals with limited population structure requires a sampling method that uniformly covers the genetic space of the breeding population as opposed to stratified sampling based on discrete classifications into sub-populations.
- When GP is implemented to lessen the resources consumed by phenotyping, a portion of the population is phenotyped to train a model that predicts the remaining individuals. The TV scheme results show the value of explicitly using genomic information to sample the training set.
- The CDmean method of selecting a training set should be utilized for genomic prediction in potato, as it is robust to sample size, trait architecture, statistical model and application scenario.
- Further investigation has to be done before these results can be extrapolated to other traits and other polyploid crops. Testing on larger pools of varieties with more distinct subgroups is required.

## Data Availability Statement

The original contributions presented in the study are included in the article/Supplementary Material, further inquiries can be directed to the corresponding author/s.

## Author Contributions

SW performed the analyses and drafted the manuscript. MM, CM, HM, and RV contributed to the discussion on analytical models and data preparation. FE guided analyses and was the general overseer for the project. All authors significantly contributed to the present study, read, and approved the final manuscript.

## Funding

This study was funded by the following sources: Solynta, Meijer Potato, Pepsico, and the Dutch Research Council (NWO).

## Conflict of Interest

The authors declare that the research was conducted in the absence of any commercial or financial relationships that could be construed as a potential conflict of interest. The handling editor declared a past collaboration with one of the authors RV.

## Publisher's Note

All claims expressed in this article are solely those of the authors and do not necessarily represent those of their affiliated organizations, or those of the publisher, the editors and the reviewers. Any product that may be evaluated in this article, or claim that may be made by its manufacturer, is not guaranteed or endorsed by the publisher.

## Appendix A: AMOVA Table to Analyse Variability Due to Population Structure

**Appendix A1:** AMOVA analysis showing sources of variation from different configurations of population structure.

**Source of variation**	**df**	**SS**	**MS**	**Est. Var**.	**Percentage**
**AMOVA with 6 market classes: | *CP* | *RWT* | *Y* | *P* | *FFP* | *TR* |**					
Among subpops	5	0.208	4.16 × 10^−2^	1.16 × 10^−3^	14.78
Within subpops	184	1.234	6.71 × 10^−3^	6.71 × 10^−3^	85.22
Total	189	1.442			100
**AMOVA with 3 market classes: | *CP, RWT, Y* | *P* | *FFP, TR* |**					
Among subpops	2	0.154	7.72 × 10^−2^	1.37 × 10^−3^	16.61
Within subpops	187	1.288	6.89 × 10^−3^	6.89 × 10^−3^	83.39
Total	189	1.442			100
**AMOVA with 3 market classes: | *CP, RWT* | *Y, P* | *FFP, TR* |**					
Among subpops	2	0.152	7.62 × 10^−2^	1.19 × 10^−3^	14.71
Within subpops	187	1.290	6.90 × 10^−3^	6.90 × 10^−3^	85.29
Total	189	1.442			100
**AMOVA with 4 market classes: | *CP, RWT* | *Y* | *P* | *FFP, TR* |**					
Among subpops	3	0.178	5.93 × 10^−2^	1.29 × 10^−3^	15.93
Within subpops	186	1.264	6.80 × 10^−3^	6.80 × 10^−3^	84.07
Total	189	1.442			100
**AMOVA with 2 market classes: | *CP* | *RWT, Y, P, FFP, TR* |**					
Among subpops	1	0.075	7.51 × 10^−2^	8.05 × 10^−4^	9.97
Within subpops	188	1.367	7.27 × 10^−3^	7.27 × 10^−3^	90.03
Total	189	1.442			100

*df, Degrees of freedom; SS, Sum of Squared deviations; MS, Mean Sum of Squared Deviations; Est. Var, Estimated Variance components; CP, Chip Processing; RWT, Round White Table; Y, Yellow (Y); P, Pigmented; FFP, French Fry Processing; TR, Table Russet. Classes grouped together between vertical lines (|)*.
