# Supplementary material for: Training Set Construction for Genomic Prediction in Auto-Tetraploids: An Example in Potato
Source: Front Plant Sci. 2021 Nov 24;12:771075. doi: 10.3389/fpls.2021.771075 (PMC8651708; doi:10.3389/fpls.2021.771075)
Supplement: Supplementary file 3 [file Table_1.DOCX]

#############################################################################

# Script to optimize the calibration set in genomic selection (maximize the expected reliability).

# Method based on the generalized CD.

# (Rincent et al. 2012)

#############################################################################

# 28/08/2012, author renaud.rincent@moulon.inra.fr

###############

#Functions used

###############

# This function creates the matrix of contrast between each of the individual not in the calibration set and the mean of the population

contrasteNonPheno=function(NotSampled_f,Nind_f,Nind_in_Sample_f)

{

mat=matrix(-1/Nind_f,Nind_f,Nind_f-Nind_in_Sample_f)

for (i in 1:ncol(mat)) {

mat[NotSampled_f[i],i]=1-1/Nind_f

}

return(mat)

}

##############################

# Data required

##########################

matA1=read.table("matA1Dent_sansPond.csv") #This is the covariance matrix betw the individuals (size Nind x xNind), estimated with the genotypes.

matA1=as.matrix(matA1)

Nind=nrow(matA1) # total number of individuals

nindrep=100 # Choose a size for your calibration set

varP=var(Pheno) # Pheno is a vector of phenotypes

h2=0.95 # Trait heritability

lambda=varE/varG # lambda is needed to estimate the CDmean

invA1=solve(matA1) # Inverse of the covariance matrix

##############################

# Optimization algo

##############################

Nind_in_Sample=nindrep

#Design matrices

Ident<-diag(Nind_in_Sample)

X<-rep(1,Nind_in_Sample)

M<-Ident- (X%*%solve(t(X)%*%X) %*% t(X) )

Sample1<-sample(Nind,Nind_in_Sample) #Calibration set initialization

SaveSample=Sample1

NotSampled1<-seq(1:Nind)

NotSampled<-NotSampled1[-Sample1] # Initial validation set

Z=matrix(0,Nind_in_Sample,Nind)

for (i in 1:length(Sample1)) { Z[i,Sample1[i]]=1 }

T<-contrasteNonPheno(NotSampled,Nind,Nind_in_Sample) # T matrice des contrastes

# Calculate of CDmean of the initial set

matCD<-(t(T)%*%(matA1-lambda*solve(t(Z)%*%M%*%Z + lambda*invA1))%*%T)/(t(T)%*%matA1%*%T)

CD=diag(matCD)

CDmeanSave=mean(CD)

CDmeanMax1=rep(NA,800)

# Exchange algorithm (maximize CDmean)

cpt2=1

cpt=0

while (cpt2<800) { # Make sure that 800 is enough in your case (that you reached a plateau), for this look at CDmeanMax1.

NotSampled=NotSampled1[-Sample1]

cpt2=cpt2+1

# Remove one individual (randomly choosen) from the sample :

Sample2=sample(Sample1,1)

# Select one individual (randomly choosen) from the individuals that are not in the Calibration set :

Sample3=sample(NotSampled,1)

# New calibration set :

Sample4=c(Sample3,Sample1[Sample1!=Sample2])

# Calculate the mean CD of the new calibration set :

Z=matrix(0,Nind_in_Sample,Nind)

for (i in 1:length(Sample4)) { Z[i,Sample4[i]]=1 }

NotSampled=NotSampled1[-Sample4]

T<-contrasteNonPheno(NotSampled,Nind,Nind_in_Sample)

matCD<-(t(T)%*%(matA1-lambda*solve(t(Z)%*%M%*%Z + lambda*invA1))%*%T)/(t(T)%*%matA1%*%T)

CD=diag(matCD)

if (mean(CD)>CDmeanSave ) { Sample1=Sample4 # Accept the new Calibration set if CDmean is increased, reject otherwise.

CDmeanSave=mean(CD)

cpt=0 } else { cpt=cpt+1

}

CDmeanMax1[cpt2-1]=CDmeanSave

} #Fin du while

SampleOptimiz=Sample1 # SampleOptimiz is the optimized calibration set

# End
