# Supplementary material for: Training Set Construction for Genomic Prediction in Auto-Tetraploids: An Example in Potato
Source: Front Plant Sci. 2021 Nov 24;12:771075. doi: 10.3389/fpls.2021.771075 (PMC8651708; doi:10.3389/fpls.2021.771075)
Supplement: Supplementary file 4 [file Table_2.DOCX]

\1.2 Genetic distances sampling

endjob

\set working directory

%cd 'D:\\...

\Define number of times you want to sample

scal ntimes; 100

\sample size

scal nsel; 200

\import a factor with the genotype names (geno)

import 'PhenoFlint.csv' ; isave=pheno

calc ng=nlev(geno)

\import the matrix with the genetic distances and convert it to Genstat matrix format

import 'A_IBS.csv' ; isave=k

calc ng=nval(k[1])

MATRIX [ROWS=ng; COLUMNS=ng] KvRs

calc nc=ng+1

CALCULATE KvRs$[*;1...ng]=k[2...nc]

\force the diagonal to have 1's (a few genotypes have a similarity slightly<1 because of heterozygous markers)

calc KvRs$[1...ng;1...ng]=1

print KvRs

SYMMETRICMATRIX [ROWS=geno] KIBS

CALCULATE KIBS=KvRs

CALCULATE mineigen=min(evalues(KIBS))

print mineigen

fspread [outf='Kmatrix_IBS.gsh'] KIBS

\spload 'Kmatrix_Euclidean.gsh'

getatt [att=lab] geno; save=labG

calc nG=nrows(KIBS)

text [nvalues=nG] geno_id

equate labG[]; geno_id

subset [cond=Env.eq.1] pheno[]

\getatt [att=lab] geno; save=geno_id

dele [rede=yes] samp

\pointer to store the samples

pointer [nval=ntimes] samp

for i= 1...100

dele [rede=yes] s2

\Generate seed for randomization

calc s=1000000* URAND(0; 1)

variate [nval=nG]samp[i]

\Sample the validation genotypes

QGSELECT [print=*;nclusters=nsel; method=samp] genotypes=geno; similarity=KIBS; selected=samp[i]; seed=s

endfor

txcons [text=gd_groups] 'GendistIBS', nsel,'genos.csv'

export [outfile=#gd_groups; csvopt=noquotes] geno_id, samp[]
